# Anesthetic Challenge in a Parturient With Von Hippel-Lindau Disease

**DOI:** 10.7759/cureus.49749

**Published:** 2023-11-30

**Authors:** Siyu Lye, Angie Phui Sze Au Yong

**Affiliations:** 1 Department of Anaesthesiology, Singapore General Hospital, Singapore, SGP

**Keywords:** anesthesia, von hippel-lindau syndrome, obstetrics, epidural, central neuraxial blockade

## Abstract

Von Hippel-Lindau (VHL) syndrome is a rare autosomal dominant disease with incomplete penetrance and variable expression. The features of cerebellar and spinal tumors, pheochromocytomas, and increased intracranial pressure complicate the anesthetic management of such patients. This report describes the anesthetic management of a parturient with VHL disease and highlights the importance of proper surveillance, vigilant management, and individualized treatment plans from a multidisciplinary team.

## Introduction

The prevalence of von Hippel-Lindau (VHL) has internationally been reported to be 1:36,000-91,000 [1]. The clinical features of the disorder include tumors or cysts arising most commonly in the cerebellum, brainstem, spinal cord, and retina. They can also be found in the adrenal glands, pancreas, kidneys, endolymphatic sac of the middle ear, broad ligament, and epididymis [2]. Anesthetists managing patients with VHL need to consider the multi-systemic involvement of the disease and carefully follow up with them throughout pregnancy, with careful surveillance to look out for potential related complications during pregnancy that may endanger the patient and fetus.

## Case presentation

A 40-year-old primipara was seen in the Singapore General Hospital High-Risk Obstetrics Pre-anaesthesia clinic at 24 weeks. She was diagnosed with type 1 VHL disease at 25 years old when she first presented with an eye infection and was diagnosed with a left optic hemangioblastoma from a CT scan. She had been closely followed up by an endocrinologist and geneticist with annual whole-body MRI surveillance scans. Her clinical features include multiple pancreatic cysts, left clear-cell papillary renal cell carcinoma (RCC), for which she underwent a partial nephrectomy in 2019, breast fibroadenoma lumps, several bilateral 0.2-0.4 cm cerebellar hemangioblastomas, and a cervicomedullary junction hemangioma with no hydrocephalus. She had no neurological symptoms and was leading an active lifestyle before conception at the time of consultation.

**Figure 1:**
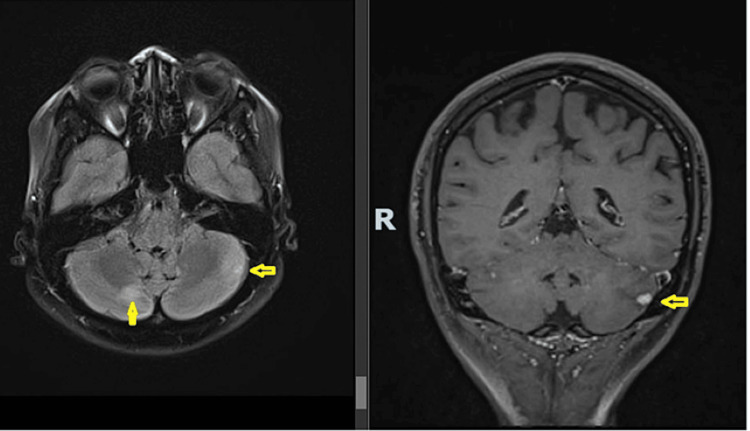
Surveillance MRI of the brain done at 20 weeks gestation, showing stable hemangioma (0.3 cm) in the cerebellar with no new mass and no features of increased intracranial pressure.

She conceived via in-vitro fertilization and her antenatal care involved a multidisciplinary team consisting of Obstetrics, Anaesthesia, Endocrinology, Genetics, and Neurosurgery. The main concern was suitability for normal vaginal delivery and the safety of central neuraxial anesthesia. Risk assessment was done with a surveillance brain MRI at 20 weeks gestation (Figure 1). It showed no new/worsening brain/spine hemangioblastomas with no hydrocephalus, mass effect, or intracranial hemorrhage. Neurosurgery deemed it safe for her to undergo both normal vaginal delivery and have spinal or epidural anesthesia as her cerebellar hemangioblastomas were small with no edema or radiological features of a tight posterior fossa, which may suggest elevated intracranial pressure. Ophthalmology confirmed no signs of papilledema. Anesthesia concurred as the hemangioblastomas were far from the site of epidural puncture at L3/4.

With clearance from the multidisciplinary team for normal vaginal delivery and neuraxial labor analgesia, the proposed delivery plan was expectant management and normal vaginal birth under epidural anesthesia. She was followed up with obstetrics routinely until delivery. Eventually, she had a vacuum-assisted normal vaginal delivery safely at 38 weeks when she presented with contractions. She opted to proceed without epidural anesthesia and birthed a 3.2 kg live male with no complications.

## Discussion

A pregnant VHL patient can be challenging for an anesthetist and requires assessment by a multidisciplinary team for various reasons: (1) Determining the mode of delivery. (2) Preventing raised intracranial pressure while straining and pushing during normal vaginal delivery. (3) The presence of cerebellum/spinal hemangiomas that may hinder the technical performance of neuraxial anesthesia.

The mode of delivery is a discussion of risks and benefits among the multidisciplinary team, and there should be shared decision-making based on the patient’s clinical condition and preferences, for example, the presence or absence of intracranial tumors, raised intracranial pressure, and pheochromocytoma [3]. The most common causes of death are complications associated with RCC and central nervous system hemangioblastomas [4]. Elevated intracranial pressure and severe hypertension during stage 2 of labor can cause rupture of hemangiomas. There is prolonged exposure to these cumulative risks during labor, which can be as long as 14 hours for a primipara [5]. If these red flags are present, it would not be unreasonable to opt for C-section instead as anesthesia would ablate these sympathetic discharges. As the patient in this case report was asymptomatic, it was reasonable to recommend normal vaginal delivery.

Next, the gold standard for labor analgesia is a lumbar epidural. This would also be excellent for mediating pain and sympathetic discharge from contractions, thereby avoiding surges in blood pressure and intracranial pressure. Most hemangioblastomas are located in the cervical and thoracic region and are within the posterior medullary cord [6]. These vascular tumors may have a mass effect or bleed if traumatized by the spinal needle or epidural catheter. It has been proposed that an epidural is preferable to spinal anesthesia as the dura is not intentionally punctured, resulting in less chance of hemangioblastoma penetration [6]. We recommend evaluating the safety of neuraxial anesthesia depending on the size and location of hemangioblastomas and the presence of raised intracranial pressure, as well as considering a plain epidural rather than a combined spinal-epidural technique. It is unclear whether pregnancy is associated with accelerated hemangioblastoma progression [7-9], but the VHL Alliance Handbook recommended a non-contrast MRI during the fourth month of pregnancy to check any known lesions of the brain and spine [10].

There is no consensus in the literature regarding the technique for obstetric anesthesia in VHL patients. Epidural, spinal, and general anesthesia have all been described in case reports with acceptable outcomes [11,12]. During general anesthesia, care should be taken to ablate the sympathoadrenergic response during intubation and surgical incision. Controlled hyperventilation can also help to control any raised intracranial pressure.

It would be worthwhile to assess risks and outline plans for both elective and emergency C-sections in case of unforeseen circumstances. The concerns and considerations should be documented so that even if the original multidisciplinary specialists are not present during non-office-hour emergencies, the emergency team is still able to achieve the intended hemodynamic targets and goals of care for the patient.

## Conclusions

A parturient with VHL presents unique challenges to the anesthetist. Early assessment by the anesthetist is recommended to allow a detailed assessment of the location and size of hemangiomas. We recommend a surveillance MRI scan in the second trimester to look for enlargement of neuraxial hemangiomas and signs of increased intracranial pressure that may affect the mode of delivery and neuraxial anesthesia. Early assessment also allows time for counseling the patient regarding the risks and benefits of each mode of delivery and labor analgesia options. Proper surveillance, vigilant management, and individualized treatment plans from a multidisciplinary team will likely have favorable maternal and fetal outcomes in this rare disorder.
